# Supplementary material for: Functional connectomics reveals general wiring rule in mouse visual cortex
Source: Nature. 2025 Apr 9;640(8058):459–69. doi: 10.1038/s41586-025-08840-3 (PMC11981947; doi:10.1038/s41586-025-08840-3)
Supplement: Supplementary file 1 — This file contains legends for Supplementary Tables 1–34. [file 41586_2025_8840_MOESM1_ESM.pdf]

nature portfolio

https://doi.org/10.1038/s41586-025-08840-3

Supplementary information

Functional connectomics reveals general wiring rule in mouse visual cortex

In the format provided by the
authors and unedited

Supplemental Information Guide

The Supplemental Tables are provided in the file **Supplemental_Tables.xlsx**. Each table is a separate sheet, with the sheet name corresponding to the table number. The first cell of each sheet contains the captions. The table numbers and captions are also listed below:

- **Supplemental Table 1**: Proofread presynaptic neuron nucleus ID's, area, layer, and proofreading strategy. nucleus_id's are from CAVE table: nucleus_detection_v0
- **Supplemental Table 2**: Pairwise comparison of the presynaptic mean in silico signal correlation between different neuron pair populations. For each comparison, a pairwise t-test was performed to test the null hypothesis that for each presynaptic neuron, the mean in silico signal correlation is the same between two postsynaptic populations. adjusted p-value is the adjusted p-value through the BH multicomparison correction procedure.
- **Supplemental Table 3**: Number of neurons and neuron pairs involved in visualizing the relationship between in silico signal correlation and L_d / neuron pair (synapses excluded) in different projection types across brain areas.
- **Supplemental Table 4**: Estimated marginal means of linear trends for the effect of in silico signal correlation on L_d / neuron pair (synapses excluded) in different projection types across brain areas. z and p-value are the z statistics and p-value of the marginal mean linear trends estimated from the fitted GLMMs. adjusted p-value is the adjusted p value through the BH multicomparison correction procedure.
- **Supplemental Table 5**: Number of neurons and neuron pairs involved in visualizing the relationship between in silico signal correlation and $N_{syn}/mm\ L_d$ in different projection types across brain areas.
- **Supplemental Table 6**: Estimated marginal means of linear trends for the effect of in silico signal correlation on $N_{syn}/mm\ L_d$ in different projection types across brain areas. z and p-value are the z statistics and p-value of the marginal mean linear trends estimated from the fitted GLMMs. adjusted p-value is the adjusted p value through the BH multicomparison correction procedure.
- **Supplemental Table 7**: Number of neurons and neuron pairs involved in the visualization of the correlation between feature weight similarity and L_d / neuron pair (synapses excluded) in different projection types across brain areas.
- **Supplemental Table 8**: Estimated marginal means of linear trends for the effect of feature weight similarity on L_d / neuron pair (synapses excluded) in different projection types across brain areas. z and p-value are the z statistics and p-value of the marginal mean linear trends estimated from the fitted GLMMs. adjusted p-value is the adjusted p value through the BH multicomparison correction procedure.
- **Supplemental Table 9**: Number of neurons and neuron pairs involved in the visualization of the correlation between receptive field center distance and L_d / neuron pair (synapses excluded) in different projection types across brain areas.
- **Supplemental Table 10**: Estimated marginal means of linear trends for the effect of receptive field center distance on L_d / neuron pair (synapses excluded) in different projection types across brain areas. z and p-value are the z statistics and p-value of the marginal mean linear trends estimated from the fitted GLMMs. adjusted p-value is the adjusted p value through the BH multicomparison correction procedure.
- **Supplemental Table 11**: Number of neurons and neuron pairs involved in the visualization of the correlation between feature weight similarity and $N_{syn}/mm\ L_d$ in different projection types across brain areas.
- **Supplemental Table 12**: Estimated marginal means of linear trends for the effect of feature weight similarity on $N_{syn}/mm\ L_d$ in different projection types across brain areas. z and p-value are the z statistics and p-value of the marginal mean linear trends estimated from the fitted GLMMs. adjusted p-value is the adjusted p value through the BH multicomparison correction procedure.

- **Supplemental Table 13**: Number of neurons and neuron pairs involved in the visualization of the correlation between receptive field center distance and $N_{syn}/mm\ L_d$ in different projection types across brain areas.
- **Supplemental Table 14**: Estimated marginal means of linear trends for the effect of receptive field center distance on $N_{syn}/mm\ L_d$ in different projection types across brain areas. z and p-value are the z statistics and p-value of the marginal mean linear trends estimated from the fitted GLMMs. adjusted p-value is the adjusted p value through the BH multicomparison correction procedure.
- **Supplemental Table 15**: Estimated marginal means of linear trends for the effect of in silico signal correlation on L_d / neuron pair (synapses excluded) in different projection types across brain areas and layers. z and p-value are the z statistics and p-value of the marginal mean linear trends estimated from the fitted GLMMs. adjusted p-value is the adjusted p value through the BH multicomparison correction procedure.
- **Supplemental Table 16**: Estimated marginal means of linear trends for the effect of in silico signal correlation on $N_{syn}/mm\ L_d$ in different projection types across brain areas and layers. z and p-value are the z statistics and p-value of the marginal mean linear trends estimated from the fitted GLMMs. adjusted p-value is the adjusted p value through the BH multicomparison correction procedure.
- **Supplemental Table 17**: Estimated marginal means of linear trends for the effect of feature weight similarity on L_d / neuron pair (synapses excluded) in different projection types across brain areas and layers. z and p-value are the z statistics and p-value of the marginal mean linear trends estimated from the fitted GLMMs. adjusted p-value is the adjusted p value through the BH multicomparison correction procedure.
- **Supplemental Table 18**: Estimated marginal means of linear trends for the effect of feature weight similarity on $N_{syn}/mm\ L_d$ in different projection types across brain areas and layers. z and p-value are the z statistics and p-value of the marginal mean linear trends estimated from the fitted GLMMs. adjusted p-value is the adjusted p value through the BH multicomparison correction procedure.
- **Supplemental Table 19**: Estimated marginal means of linear trends for the effect of receptive field center distance on L_d / neuron pair (synapses excluded) in different projection types across brain areas and layers. z and p-value are the z statistics and p-value of the marginal mean linear trends estimated from the fitted GLMMs. adjusted p-value is the adjusted p value through the BH multicomparison correction procedure.
- **Supplemental Table 20**: Estimated marginal means of linear trends for the effect of receptive field center distance on $N_{syn}/mm\ L_d$ in different projection types across brain areas and layers. z and p-value are the z statistics and p-value of the marginal mean linear trends estimated from the fitted GLMMs. adjusted p-value is the adjusted p value through the BH multicomparison correction procedure.
- **Supplemental Table 21**: Paired t-tests for comparing the mean presyn-postsyn functional similarity between observation in the MICrONS dataset and values expected by GLMMs fit on the MICrONS dataset
- **Supplemental Table 22**: Paired t-tests for comparing the mean postsyn-postsyn functional similarity between observation in the MICrONS dataset and values expected by GLMMs fit on the MICrONS dataset
- **Supplemental Table 23**: Number of neurons and neuron pairs involved in visualizing the relationship between in vivo signal correlation and L_d / neuron pair (synapses excluded) in different projection types across brain areas.
- **Supplemental Table 24**: Estimated marginal means of linear trends for the effect of in vivo signal correlation on L_d / neuron pair (synapses excluded) in different projection types across brain areas. z and p-value are the z statistics and p-value of the marginal mean linear trends estimated from the fitted GLMMs. adjusted p-value is the adjusted p value through the BH multicomparison correction procedure.
- **Supplemental Table 25**: Number of neurons and neuron pairs involved in visualizing the relationship between in vivo signal correlation and $N_{syn}/mm\ L_d$ in different projection types across brain areas.
- **Supplemental Table 26**: Estimated marginal means of linear trends for the effect of in vivo signal correlation on $N_{syn}/mm\ L_d$ in different projection types across brain areas. z and p-value are the z statistics and p-value of the marginal mean linear trends estimated from the fitted GLMMs. adjusted p-value is the adjusted p value through the BH multicomparison correction procedure.

- **Supplemental Table 27**: Estimated marginal means of linear trends for the effect of in vivo signal correlation on L_d / neuron pair (synapses excluded) in different projection types across brain areas and layers. z and p-value are the z statistics and p-value of the marginal mean linear trends estimated from the fitted GLMMs. adjusted p-value is the adjusted p value through the BH multicomparison correction procedure.
- **Supplemental Table 28**: Estimated marginal means of linear trends for the effect of in vivo signal correlation on $N_{syn}/mm\ L_d$ in different projection types across brain areas and layers. z and p-value are the z statistics and p-value of the marginal mean linear trends estimated from the fitted GLMMs. adjusted p-value is the adjusted p value through the BH multicomparison correction procedure.
- **Supplemental Table 29**: Number of neurons and neuron pairs involved in the visualization of the correlation between in silico ΔOri and L_d / neuron pair (synapses excluded) in different projection types across brain areas.
- **Supplemental Table 30**: Estimated marginal means of linear trends for the effect of in silico ΔOri on L_d / neuron pair (synapses excluded) in different projection types across brain areas. z and p-value are the z statistics and p-value of the marginal mean linear trends estimated from the fitted GLMMs. adjusted p-value is the adjusted p value through the BH multicomparison correction procedure.
- **Supplemental Table 31**: Number of neurons and neuron pairs involved in the visualization of the correlation between in silico ΔOri and $N_{syn}/mm\ L_d$ in different projection types across brain areas.
- **Supplemental Table 32**: Estimated marginal means of linear trends for the effect of in silico ΔOri on $N_{syn}/mm\ L_d$ in different projection types across brain areas. z and p-value are the z statistics and p-value of the marginal mean linear trends estimated from the fitted GLMMs. adjusted p-value is the adjusted p value through the BH multicomparison correction procedure.
- **Supplemental Table 33**: Estimated marginal means of linear trends for the effect of in silico ΔOri on L_d / neuron pair (synapses excluded) in different projection types across brain areas and layers. z and p-value are the z statistics and p-value of the marginal mean linear trends estimated from the fitted GLMMs. adjusted p-value is the adjusted p value through the BH multicomparison correction procedure.
- **Supplemental Table 34**: Estimated marginal means of linear trends for the effect of in silico ΔOri on $N_{syn}/mm\ L_d$ in different projection types across brain areas and layers. z and p-value are the z statistics and p-value of the marginal mean linear trends estimated from the fitted GLMMs. adjusted p-value is the adjusted p value through the BH multicomparison correction procedure.
