# Supplementary material for: Functional connectomics reveals general wiring rule in mouse visual cortex
Source: Nature. 2025 Apr 9;640(8058):459–69. doi: 10.1038/s41586-025-08840-3 (PMC11981947; doi:10.1038/s41586-025-08840-3)
Supplement: Supplementary file 2 — Reporting Summary [file 41586_2025_8840_MOESM2_ESM.pdf]

nature portfolio

Corresponding author(s): Andreas Tolias

Last updated by author(s): 2/13/2025

Reporting Summary

Nature Portfolio wishes to improve the reproducibility of the work that we publish. This form provides structure for consistency and transparency in reporting. For further information on Nature Portfolio policies, see our Editorial Policies and the Editorial Policy Checklist.

Statistics

For all statistical analyses, confirm that the following items are present in the figure legend, table legend, main text, or Methods section.

n/a	Confirmed	
☐	☒	The exact sample size (*n*) for each experimental group/condition, given as a discrete number and unit of measurement
☐	☒	A statement on whether measurements were taken from distinct samples or whether the same sample was measured repeatedly
☐	☒	The statistical test(s) used AND whether they are one- or two-sided<br>*Only common tests should be described solely by name; describe more complex techniques in the Methods section.*
☐	☒	A description of all covariates tested
☐	☒	A description of any assumptions or corrections, such as tests of normality and adjustment for multiple comparisons
☐	☒	A full description of the statistical parameters including central tendency (e.g. means) or other basic estimates (e.g. regression coefficient) AND variation (e.g. standard deviation) or associated estimates of uncertainty (e.g. confidence intervals)
☐	☒	For null hypothesis testing, the test statistic (e.g. *F*, *t*, *r*) with confidence intervals, effect sizes, degrees of freedom and *P* value noted<br>*Give P values as exact values whenever suitable.*
☒	☐	For Bayesian analysis, information on the choice of priors and Markov chain Monte Carlo settings
☒	☐	For hierarchical and complex designs, identification of the appropriate level for tests and full reporting of outcomes
☐	☒	Estimates of effect sizes (e.g. Cohen's *d*, Pearson's *r*), indicating how they were calculated

Our web collection on statistics for biologists contains articles on many of the points above.

Software and code

Policy information about availability of computer code

Data collection	For image acquisition, we used ScanImage (2017b). Stimuli were presented using PsychToolBox (3). The data collection process was automated with Labview (2016).
Data analysis	We used DeepLabCut (2.0.5) for automatic tracking of the pupil. We used CaImAn (1.0) for segmentation / deconvolution of calcium imaging data. Meshparty (1.16), NEURD (1.0.0), pcg_skel (0.3, 0.2) and Neuroglancer (https://github.com/seung-lab/neuroglancer) were used for morphology analysis and visualization. Our custom built analysis pipeline (https://github.com/cajal/pipeline, developed in Matlab (2016a, 2018b), Python (3.6, 3.8), and R (4.3.3)) also used general tools like Numpy (1.23.5), pandas (1.5.3), SciPy (1.10.1), PyTorch (1.12.1), Matplotlib (3.7.0), seaborn (0.12.2), HoloViews (1.15.4), Ipyvolume (0.5.2), tidyverse (2.0.0), Jupyter (ipykernel: 6.21.2), MySQL (5.7.37), Docker (23.0.1), and Kubernetes (1.22.11). We used statsmodels (0.13.5), scikit-learn (1.2.1), glmmTMB (1.1.10), performance (0.12.2), emmeans (1.10.3) for statistical analysis. DataJoint (0.12.9) and CAVE (4.12, 4.14, 4.16) were used for storing and managing data.

For manuscripts utilizing custom algorithms or software that are central to the research but not yet described in published literature, software must be made available to editors and reviewers. We strongly encourage code deposition in a community repository (e.g. GitHub). See the Nature Portfolio guidelines for submitting code & software for further information.

Data

Policy information about availability of data

All manuscripts must include a data availability statement. This statement should provide the following information, where applicable:

- Accession codes, unique identifiers, or web links for publicly available datasets
- A description of any restrictions on data availability
- For clinical datasets or third party data, please ensure that the statement adheres to our policy

All MICrONS data are released on BossDB (https://bossdb.org/project/microns-minnie, please also see https://www.microns-explorer.org/cortical-mm3 for details).

Human research participants

Policy information about studies involving human research participants and Sex and Gender in Research.

Reporting on sex and gender	N/A
Population characteristics	N/A
Recruitment	N/A
Ethics oversight	N/A

Note that full information on the approval of the study protocol must also be provided in the manuscript.

Field-specific reporting

Please select the one below that is the best fit for your research. If you are not sure, read the appropriate sections before making your selection.

☒ Life sciences ☐ Behavioural & social sciences ☐ Ecological, evolutionary & environmental sciences

For a reference copy of the document with all sections, see nature.com/documents/nr-reporting-summary-flat.pdf

Life sciences study design

All studies must disclose on these points even when the disclosure is negative.

Sample size	No sample-size calculation was performed a priori. Sample sizes (number of connections tested) match or exceed previous studies of similar design.
Data exclusions	Of the 14 released MICrONS scans, one scan was excluded a priori from the study due to experimental issues (responses to some stimuli were not collected due to water running out from the objective). Neurons that did not pass the pre-established functional thresholds described in the paper were excluded from the analysis in order to only compare functional properties in neurons that were well characterized.
Replication	Due to the cost and time involved in producing the MICrONS volume, a second volume is not yet prepared to allow reproducibility testing. All available proofreading in the existing volume was used in order to increase power, especially where the number of unique presynaptic neurons was the limiting factor.
Randomization	No randomization is performed since our study did not include multiple predefined experimental groups for sample allocation. Instead, each sample was controlled with a matched control population ("same region" and "ADP" controls, as described in the study) with matched anatomical properties at the appropriate scale.
Blinding	No blinding is performed during data collection since our study did not include predefined experimental groups for sample allocation. Manual annotation of the data is blinded to the functional properties of the neuron. The analysis is performed unblinded, however, the same process is applied to all control and sample groups.

Reporting for specific materials, systems and methods

We require information from authors about some types of materials, experimental systems and methods used in many studies. Here, indicate whether each material, system or method listed is relevant to your study. If you are not sure if a list item applies to your research, read the appropriate section before selecting a response.

Materials & experimental systems

n/a	Involved in the study
☒	☐ Antibodies
☒	☐ Eukaryotic cell lines
☒	☐ Palaeontology and archaeology
☐	☒ Animals and other organisms
☒	☐ Clinical data
☒	☐ Dual use research of concern

Methods

n/a	Involved in the study
☒	☐ ChIP-seq
☒	☐ Flow cytometry
☒	☐ MRI-based neuroimaging

Animals and other research organisms

Policy information about studies involving animals; ARRIVE guidelines recommended for reporting animal research, and Sex and Gender in Research

Laboratory animals	For experiments excluding the MICrONS dataset in this manuscript: ten mice, (Mus musculus, 3 female, 7 males) 78-190 days old at first experimental scan. Heterozygous for both Slc17a7-Cre (B6;129S-Slc17a7tm1.1(cre)Hze/J, Jackson Laboratory Strain # 023527) and Ai162 (B6.Cg-Igs7tm162.1(tetO-GCaMP6s,CAG-tTA2)Hze/J, Jackson Laboratory Strain # 031562). The MICrONS dataset was collected from a mouse of the same species and strain, 75 days old.
Wild animals	Study did not involve wild animals.
Reporting on sex	For new experiments in this manuscript: 3 Female, 7 Males. For MICrONS dataset, 1 Male. Animals were randomly recruited to the study with respect to sex. Analysis disaggregated for sex was not performed, due to low sample size and expected generalization of principles under study across genders.
Field-collected samples	Study did not involve samples collected from the field.
Ethics oversight	All procedures were approved by the Institutional Animal Care and Use Committee of Baylor College of Medicine.

Note that full information on the approval of the study protocol must also be provided in the manuscript.
